# An Insight into the Changes in Human Plasma Proteome on Adaptation to Hypobaric Hypoxia

**DOI:** 10.1371/journal.pone.0067548

**Published:** 2013-07-02

**Authors:** Yasmin Ahmad, Narendra K. Sharma, Iti Garg, Mohammad Faiz Ahmad, Manish Sharma, Kalpana Bhargava

**Affiliations:** 1 Peptide and Proteomics Division, DIPAS, DRDO, Ministry of Defence, Delhi, India; 2 Department of Genomics, DIPAS, DRDO, Ministry of Defence, Delhi, India; 3 Department of Chemistry, Jamia Millia Islamia, New Delhi, India; Aligarh Muslim University, India

## Abstract

Adaptation to hypobaric hypoxia is required by animals and human in several physiological and pathological situations. Hypobaric hypoxia is a pathophysiological condition triggering redox status disturbances of cell organization leading, *via* oxidative stress, to proteins, lipids, and DNA damage. Identifying the molecular variables playing key roles in this process would be of paramount importance to shed light on the mechanisms known to counteract the negative effects of oxygen lack. To obtain a molecular signature, changes in the plasma proteome were studied by using proteomic approach. To enrich the low-abundance proteins in human plasma, two highly abundant proteins, albumin and IgG, were first removed. By comparing the plasma proteins of high altitude natives with those of a normal control group, several proteins with a significant alteration were found. The up-regulated proteins were identified as vitamin D-binding protein, hemopexin, alpha-1–antitrypsin, haptoglobin β-chain, apolipoprotein A1, transthyretin and hemoglobin beta chain. The down-regulated proteins were transferrin, complement C3, serum amyloid, complement component 4A and plasma retinol binding protein. Among these proteins, the alterations of transthyretin and transferrin were further confirmed by ELISA and Western blotting analysis. Since all the up- and down- regulated proteins identified above are well-known inflammation inhibitors and play a positive anti-inflammatory role, these results show that there is some adaptive mechanism that sustains the inflammation balance in high altitude natives exposed to hypobaric hypoxia.

## Introduction

The stress at high altitude is hypobaric hypoxia resulting from the lowered barometric pressure. It is unavoidable, un-modifiable, and uniform for everyone at any given altitude. Organisms at higher altitudes must adapt to the stress of limited oxygen availability relative to sea level and still be able to sustain aerobic metabolic processes. The reduced partial pressure of oxygen triggers the onset of an adaptive response, aimed at increasing the local oxygen concentration by several complementary actions [1]. Hypoxic stress impinges on well-characterized physiological pathways related to oxidative energy metabolism, which has facilitated the identification of high-altitude adaptation mechanisms in nonhuman animals [2], [3]. While many facets of hypoxic adaptation, particularly those driven by HIF-1, have come to light over the past decade, much of this work has been carried out in cultured cells and animal models. HIF was identified originally by its binding to a hypoxia response element in the human erythropoietin gene [4], [5]. Subsequently, hypoxia response elements containing HIF-1 binding sites were identified in genes encoding transferrin [6], vascular endothelium growth factor (VEGF) [7], [8], inducible nitric oxide synthase (iNOS) [9], glucose transporter 1 (GLUT 1) [10], and several glycolytic enzymes, all playing important roles in systemic, tissues, or intracellular O_2_ homeostasis allowing for increased anaerobic ATP synthesis. Some molecular variables involved in the defense against hypobaric hypoxia were identified in animal models and at cellular level [10], [11].

The integrated response to hypoxic challenge in man is much less well understood and a number of controversies exist regarding the timings of such adaptations, the degree of the hypoxia in which they occur, and the tissue specificity of alterations in gene expression and metabolism. The emergence of new technologies that enable relatively inexpensive, comprehensive and high-throughput analysis of gene expression, protein levels, and metabolic markers has the potential to contribute much to this area of research. Currently, very few investigators have applied these technologies to the study of humans at altitude and in hypoxia chambers, and only then under a limited number of hypoxic conditions and durations, and with small sample sizes. Recent advances in proteomic techniques make it possible to monitor plasma protein expression profiles providing a better insight into the mechanisms involved in functional adaptations of cells, tissues, organs, and the whole organism in the hypoxic environment. Indeed, this approach allows identifying dysregulated proteins as well as the underlying signalling involved. In addition, it could shed light on the understanding of some crucial events characterizing cell proliferation and apoptosis.

Man exposed to high altitude hypoxia is a convenient model for these investigations particularly when undergoing proteome analysis. The advantage of a proteomic rather than a transcriptomic approach is that protein expression levels are measured directly, rather than being inferred from abundance of the corresponding mRNAs, which are imperfectly correlated to protein concentration [12], [13] because of variable rates of synthesis and differences in message stability [14]. In fact, it allows identifying among several thousand proteins in plasma the molecular players undergoing significant changes as a function of a set of important variables of physiological interest (such as duration and degree of hypoxia, acid–base imbalance, nutritional habits, training, and exercise levels ) known to influence the profiles of general and local oxygen partial pressure.

In this study, the changes in plasma proteome of high altitude natives and healthy control individuals were compared by 2-DE and MS. The results showed that most of the plasma proteins found in high altitude natives are acute phase proteins (APPs), compliment components and apolipoproteins and so on. These molecular variables may play important roles in the adaptative process that could be of paramount importance to shed light on the mechanisms known to counteract the negative effects of oxygen lack.

## Materials and Methods

### Collection and Isolation of Plasma Samples

Proteomic analysis was performed on plasma samples of randomly selected high altitude natives male (n = 10) and compared with healthy male sea level controls (n = 10). The plasma from high altitude natives (ages 25–30 yrs) who were born and brought up at an altitude between 3500 and 4000 m were collected at Defence Institute of High Altitude Research (DIHAR, Leh, India). The plasma from the healthy sea level controls were collected in Defence Institute of Physiology and Allied Sciences (DIPAS, Delhi, India). Prior to the present experiment they had not been exposed to altitude. The control groups were non-smokers, ages 25–30 yrs. Volunteers were provided details of the study design as approved by the Ethical Committee of Defence Institute of Physiology and Allied Sciences and written consent was obtained. During the study period all volunteers from both the groups were provided identical food as per the standard Army ration scale (energy intake: 3200 to 3400 kcal). Fasting venous blood samples were collected from an antecubital vein in EDTA –treated vials in the morning (0800 to 0900_ AM_) at sea level and kept at 4°C until preparation to prevent coagulation and minimize protein degradation. Blood samples of high altitude natives were collected only at high altitude following the same procedure. The specimens were then centrifuged at 1500 g for 10 mins/4°C. Supernatants were transferred to new tubes as aliquots. To each 1.0 mL plasma aliquots, 10 µl of protease inhibitor were added to obtain the reproducible results by 2DE analysis. The plasma samples were stored in −80°C until the assay. All samples used in this study were prepared within 1 hour of sample collection and showed no signs of hemolysis.

### Depletion of Albumin and IgG

Because albumin and immunoglobulin IgG collectively account for approx 70% of the total plasma protein content [15], we selectively removed these proteins to enrich for proteins of lower abundance. A dye-based Proteoprep blue albumin and IgG depletion kit (Sigma Aldrich, Germany) was used according to the manufactureŕs instructions. Briefly, the provided suspended slurry medium were added to the spin columns, centrifuged and equilibrated at 8000 g for 10 seconds. The spin columns were collected in fresh collection tube. To each spin column 0.1 mL of plasma sample were added to the packed medium bed, incubated for 10 minutes, centrifuged at 8000 g for 60 seconds, repeated the same step twice to remove the additional albumin. The two times depleted plasma were remained in the collection tube and pooled for optimal protein recovery. The albumin/IgG depleted plasma samples were stored at −80°C for long-term storage.

### SDS-PAGE Analysis

Sodium dodecylsulfate-polyacrylamide gel electrophoresis (SDS-PAGE) analysis was carried out with the Tris/glycine buffer system according to Laemmli [16]. Two microlitres (20 µg) albumin and IgG depleted proteins of healthy sea level controls and HAPE patients were separated under reducing conditions on 12% SDS-PAGE mini gels (10×10.5 cm) at 250 V, 40 mA, and constant currents for 2 hours and visualized by colloidal Comassie Blue G-250 or Silver staining according to standard protocols. The gels were scanned in Ultra Lum Omega 16Vs system.

### Acetone/TCA Precipitation

A 100 µl of plasma sample was diluted with 900 µl of 10% TCA in acetone. The mixture was incubated overnight at −20°C and centrifuged at 15 000 g, 4°C for 10 min. The supernatant was removed and 1.0 mL of 90% ice-cold acetone was added to wash the pellet. The sample was incubated at −20°C for 10 min and centrifuged as above. The acetone containing supernatant was removed and the pellet was air dried. For 2D gel electrophoresis, the protein pellet was suspended in 100 µl of lysis buffer, as described earlier [17]. The protein sample was stored frozen at −20°C until analysis.

### First-dimensional IEF Using the Protean IEF Cell

Total protein content in plasma samples was determined by Bradford assay and employed bovine albumin standards. Immobilized linear pH gradient strips (17 cm, pH 5–8, Biorad ) were rehydrated with the individual plasma samples, 500 µg of protein, in 300 µl of a improved rehydration buffer solution as described earlier [18] containing 7 M urea, 2 M thiourea, 1.2% CHAPS (w/v), 0.4% ABS-14 (w/v), 20 mM dithiothreitol (DTT), 0.25% ampholytes (v/v; pH 3–10) and 0.005% bromophenol blue (BPB) (w/v), for 18 hours without current (in-gel passive rehydration). After rehydration, the focusing tray was renewed to remove any proteins not absorbed into the strip. IEF was conducted using a Protean IEF Cell (Bio-Rad) at 20°C as follow: 250 V for 1 hour (slow ramping), changing the wicks every 30 min (to assist removal of ionic contaminants), 1000 V for 1 hour, linear ramping 10,000 V to over 3 hours and a constant of 10,000 V until approximately 60 kVh was reached. Strips were removed and stored at –80°C until run on the second dimension.

### Second-dimensional Electrophoresis

For 2-DE analysis, individual samples (n = 10) were repeated at minimum in triplicates. Prior to SDS-PAGE, the IPG strips were equilibrated twice for 15 min with gentle shaking. The first equilibration solution contained 50 mM Tris-HCl (pH 8.8), 6 M urea, 30% glycerol (v/v), 2% SDS (w/v), 1% DTT (w/v) and 0.01% BPB (w/v). In the second equilibration solution, DTT was replaced with 2.5% iodoacetamide (w/v). The equilibrated IPG strips were slightly rinsed with milli-Q water, blotted to remove excess equilibration buffer and then applied to SDS-PAGE gels (20 cm×20 cm×1 mm 8–19% polyacrylamide acrylamide (30% w/v): bis-acrylamide (0.8% w/v), 37.5∶1 stock) using a PROTEAN II XL system (Bio Rad) at 10 mA per gel for 30 min followed by 35 mA per gel for 12 hours until the dye front had run off the edge of the 2-D gel.

### Staining and Imaging

After electrophoresis, proteins were visualized by modified silver staining procedure compatible with MS [19]. The gels were fixed in methanol (50% v/v), acetic acid (12% v/v) and formaldehyde (0.05% v/v) for at least 2 hours. The fixed gels were rinsed with ethanol (50% v/v) thrice for 20 min, then again sensitized with sodium thiosulfate (0.02% w/v )followed by three washings with milli-Q water each for 20 s. The gels were immersed in silver nitrate (0.1% w/v) and formaldehyde (0.075% v/v) for 20 min and rinsed with milli-Q water twice for 20 s each. It was developed with sodium carbonate (6%) and formaldehyde (0.05% v/v). Finally, the reaction was terminated by fixing with methanol (50% v/v) and acetic acid (12% v/v). The stained gels were imaged using an Investigator ™ ProPic II Genomics Solutions and the analysis of digitized images with Image Master 2D Platinum v.6 software (GE Healthcare). Automatic spot detection and matching of the gels was done, followed by manual rechecking of the matched and unmatched protein spots. The intensity volumes of the individual spots were normalized with the total intensity volume of all the spots present in each gel (%V). The principles of measuring intensity values by 2D analysis software are similar to those of densitometric measurement. After the background subtraction the protein spots were automatically defined and quantified with the feature detection algorithm. Spot intensities were expressed as relative volumes in percentages (% volume) by integrating the OD of each pixel in the spot area (vol) and dividing with the sum volumes of all spots detected in the gel. One of the gels was selected as a reference gel to which each other gel used in the analysis was aligned and matched, as described in the manual, using landmarks. In the reference gel each spot is assigned a unique number. The quality of the match made by the computer was critically evaluated in each case, and necessary editions and corrections were done manually. Initially, protein spots with significant changes test (paired t-test, p ? 0.05) in a consistent direction (increase or decrease) were cut for identification, according to the method of Turko et al. [20]. Comparisons were made between pairs of groups with three gels in each group. Normally, only spots that are statistically different between control and experimental groups were subjected to in-gel trypsin digestion for subsequent analysis by mass spectrometry. Each sample was run in triplicate to reduce variability and increase confidence in the resultant protein identification.

## MS Identification of Proteins

### In-gel Digestion with Trypsin and Extraction of Peptides

The procedure for in-gel digestion of protein spots from silver stained gels was performed. In brief, protein spots were extensively washed with ultrapure water and each gel spot was excised with a clean scalpel. The spots were destained and incubated for 30 min with 30 mM Potassium ferricyanide and 100 mM sodium thiosulfate at room temperature. The gel pieces were rinsed several times with water to remove destaining solution. The gel pieces were washed for 15 min at room temperature with water and 50 mM NH_4_HCO_3_/Acetonitrile. Enough acetonitrile were added to cover gel pieces for shrinking the gel pieces. The gel pieces were rehydrated in 10 mM NH_4_HCO_3_ for 5 min, equal volume of acetonitrile were added and removed after 15 min of incubation. The gel pieces were again covered with acetonitrile and removed. The gel pieces were dried in a vacuum centrifuge. The dried gel pieces were digested with 20 µl of trypsin (20 ng/µl, Trypsin Singles™ Proteomics Grade, Sigma) and incubated the sample at 37°C overnight, the tryptic peptide were sonicated for 10 min and dried in a speed Vac. The dried peptides were extracted with 5 µl of 0.1% TFA.

### MALDI-TOF/TOF

For PMF, in-gel tryptic peptides of each spot of interest were mixed with an acidic solid matrix such as α-cyano-4-hydroxy cinnamic acid (CHCA) matrix 10 mg/ml, which provides high sensitivity and negligible matrix adduction during the laser absorption and subjected to laser radiation. The matrix was made in 70% acetonitrile and 0.03% TFA. 0.5 µl of the peptide extracts mixed with the 0.5 µl of the matrix were manually spotted onto a 600 µm/384 well AnchorChip™ sample target (Bruker Daltonics) and dried at ambient temperature. Peptide mass spectra were recorded in the reflectron mode using an Ultraflex III Tof/Tof mass spectrometer (Bruker Daltonics) equipped with a 384-sample scout source. The ion acceleration voltage after pulsed extraction was 29,000 V. A peptide calibration standard (Bruker Daltonics) was used for external calibration as described previously [21]. MS and MS/MS data were recorded automatically on the MALDI-TOF/TOF instrument using the three most abundant peptide signals of the corresponding peptide mass fingerprint (PMF) spectrum. The monoisotopic peak list was generated in Post Processing s/w and True peptide mass list was generated by Bruker Flex Analysis software version 3.0 and Biotools ver 3.1 without using the smoothing function and the peak filter was applied to exclude the masses lower than 700 Da and the signal to noise ratio of 20. The generated peptide mass list was sent for the online data base search to find and match the Protein Identity. The search engine “MASCOT Server” (www.matrixscience.com) was used to obtain the protein identity by undertaking the Peptide Mass Fingerprinting approach. Databases searches were performed taking into account carbamidomethyl modification of cysteines and possible oxidation of methionine. One missed cleavage was allowed. A mass accuracy of ≤100 ppm was requested for PMF. For MS/MS searches, a mass accuracy of ≤70 ppm was allowed for peptide masses and their fragments, respectively. Search was performed in NCBInr, MSDB and SwissProt database with the following search parameter: Mass Tolerance: 50 ppm to 100 ppm; species, Homo sapience; maximum number of missed cleavages was set to 1 for all samples. Once the protein was identified, the identity was confirmed by using Tandem Mass spectrometry. For each identified Protein, at least one Peptide was selected for MS/MS (TOF/TOF) to validate the Protein Identity. Instrument was used in the Lift mode (TOF/TOF) to obtain the MS/MS spectra. Again the Flex Analysis 3.0 and Biotools 3.1 s/w were used to generate the fragments mass list and the sequence Tag of peptide. The mass list was sent to database in same way as was done in case of above PMF approach. The mass tolerance error of 0.5 Da to 1 Da was used for MS/MS ion search. The MS/MS ion search confirmed the protein identity and provided the amino acid sequence of particular peptide. Gene ontology (GO) annotations (functional distribution) for identified proteins were assigned using toppgene suite (http://toppgene.cchmc.org). A corresponding gene list was created from the list of proteins which were differentially expressed in high altitude natives to identify the most significant biological functions. A p value less than p≤0.05 were considered to be significant [22].

### Quantitative Validation by Enzyme-linked Immunosorbent Assay

To determine the correlation of transferrin and transthyretin with high altitude native, 20 plasma samples, including 10 high altitude natives and 10 from normal control group were used for quantitative validation. The total transferrin and transthyretin were quantified by using competitive ELISA kit with the purified polyclonal antibody against transferrin and transthyretin (ICL, USA) according to the manufactureŕs instructions. Briefly individual plasma samples were diluted with mix diluent (1∶40,000 dilutions for transferrin and 1∶10,000 for transthyretin). The diluted mixtures of 100 µl were added onto 96-well plate and immediately incubated at RT (30 min for transferrin and 60 min for transthyretin). After four washes with buffer, 100 µl of enzyme antibody conjugate were added and incubated for 30 min at RT in dark. After five washes with buffer, 100 µl of chromogen substrate were added and incubated for 10 min till the optimal blue color density developed. A 100 µl of stop solution were added to each well and yellow color developed and measured absorbance on a microplate reader at a wavelength of 450 nm immediately.

### Quantitative Validation by Western Blot Analysis

The protein quantification of transferrin and transthyretin were selected to be validated by western-blot analysis on the basis of their expression and significance. Secondly, obtaining of their antibodies was convenient. Briefly, plasma samples were first diluted 10 times by 1×PBS, and then total proteins (40 µg) were separated by SDS-PAGE and electro-blotted to nitrocellulose membrane. After being blotted with 5% nonfat-dried milk in 1×TBST (25 mM Tris (pH 7.5), 150 mM NaCl, 0.1% Tween 20) overnight, membranes were incubated with primary antibodies for 2 hours, followed by secondary antibody for another hour. All these experiments were conducted at room temperature. The immunocomplexes were visualized by chemiluminescence using the chemiluminescent peroxidase substrate kit (Sigma-Aldrich, St. Louis Mo 63103, USA). The film signals were digitally scanned and then quantified using image J software.

### Statistical Analysis

Statistical analyses of proteomic data were performed automatically by the Image Master 2D Platinum v.6 software (GE Healthcare). The results of ELISA and western blots are representations of three separate experiments (Mean ± SEM). Statistical analysis of ELISA and western blots were done using GraphPad Prism (Version 5) and a p value of a p-value of <0.05 was considered significant.

## Results

### Removal of High Abundance Proteins from Plasma and Acetone/TCA Precipitation

**Figure 1:**
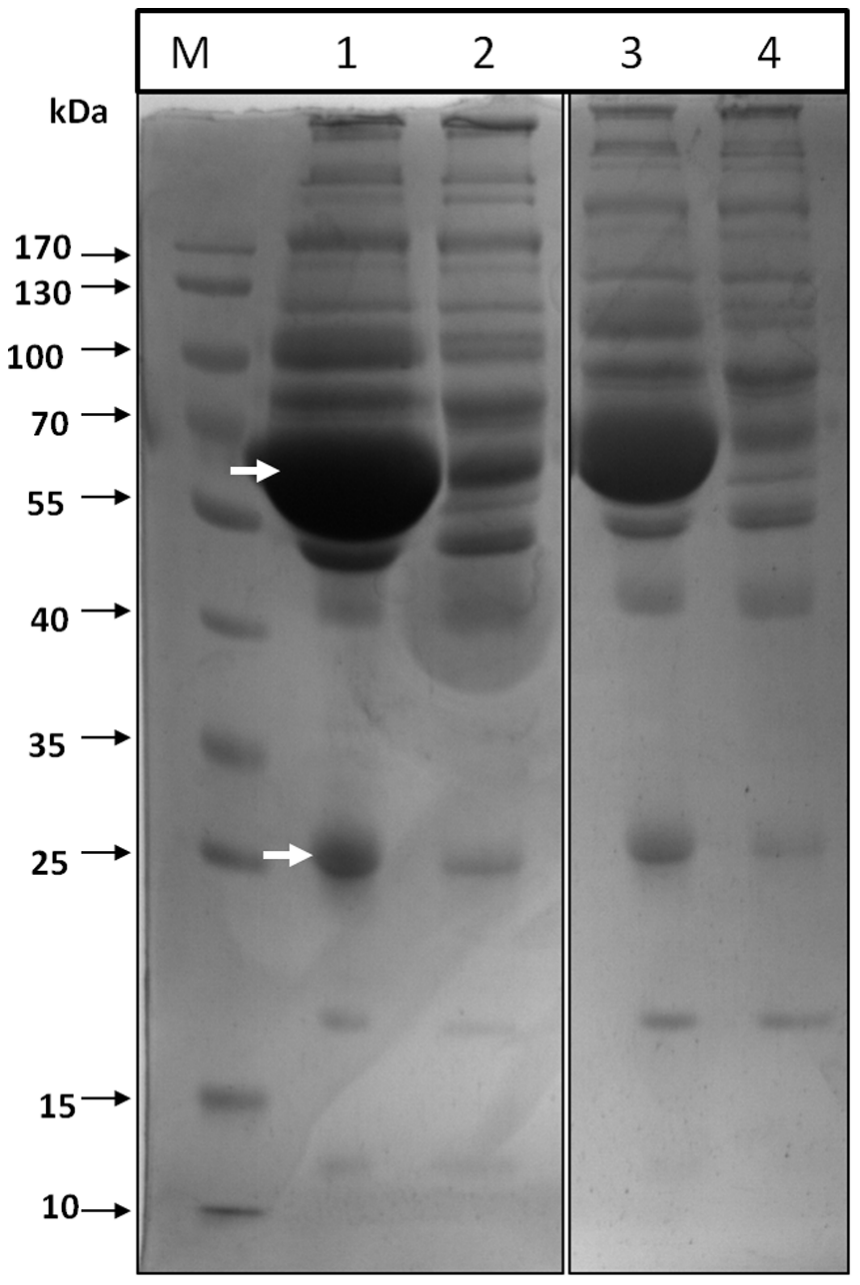
Representative SDS-PAGE image of depleted human plasma sample from control and high altitude native (HAN). Lane 1 and 3 represent crude plasma samples of control and HAN, respectively. Lane 2 and 4 represent depleted plasma samples of control and HAN, respectively. Lane M represents molecular weight marker.

Because albumin and IgG account for 80% proteins in human plasma, they are a bar to 2-DE analysis. Removing the high-abundance proteins from plasma samples can increase the visibility of low-abundance proteins and enable precise analysis. We selected to remove the high abundance proteins before 2-DGE, and the effect of depletion were evaluated by SDS-PAGE, and a good efficiency was shown (Figure 1). Depletion of human plasma resulted in significant removal of albumin at 64 kDa and IgG bands at 50 kDa and 25 kDa (Lane 2 and 4) with no apparent loss of other proteins in both the groups compared to undepleted plasma (Lane 1 and 3). Concomitant with the removal of albumin there was a significant enhancement of the staining of several protein spots as observed in two dimensional gel profiles in control and high altitude native group. We carried out acetone/TCA precipitation for the removal of protease activity, biological contaminants and enrichment of proteins.

### Proteome Profiles of High Altitude Native in Comparison with Sea Level Control

**Figure 2:**
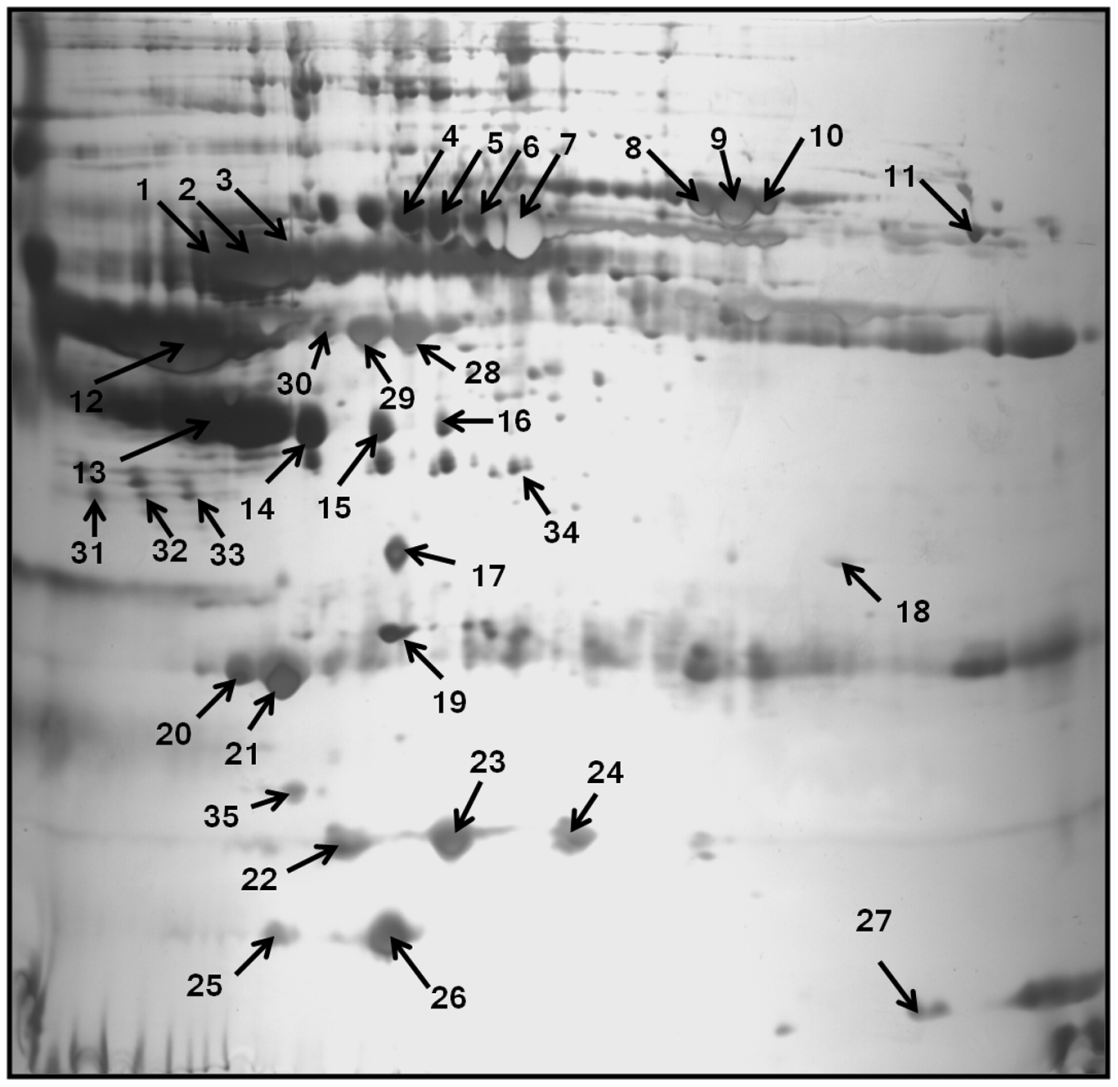
Representative gel image of plasma proteins from HAN. The proteins were resolved according to their isoelectric point (pI) in the first (5–8 pH) and their Mw on 12% SDS-PAGE followed by silver staining. Numbers mark protein spots were differentially expressed.

**Figure 3:**
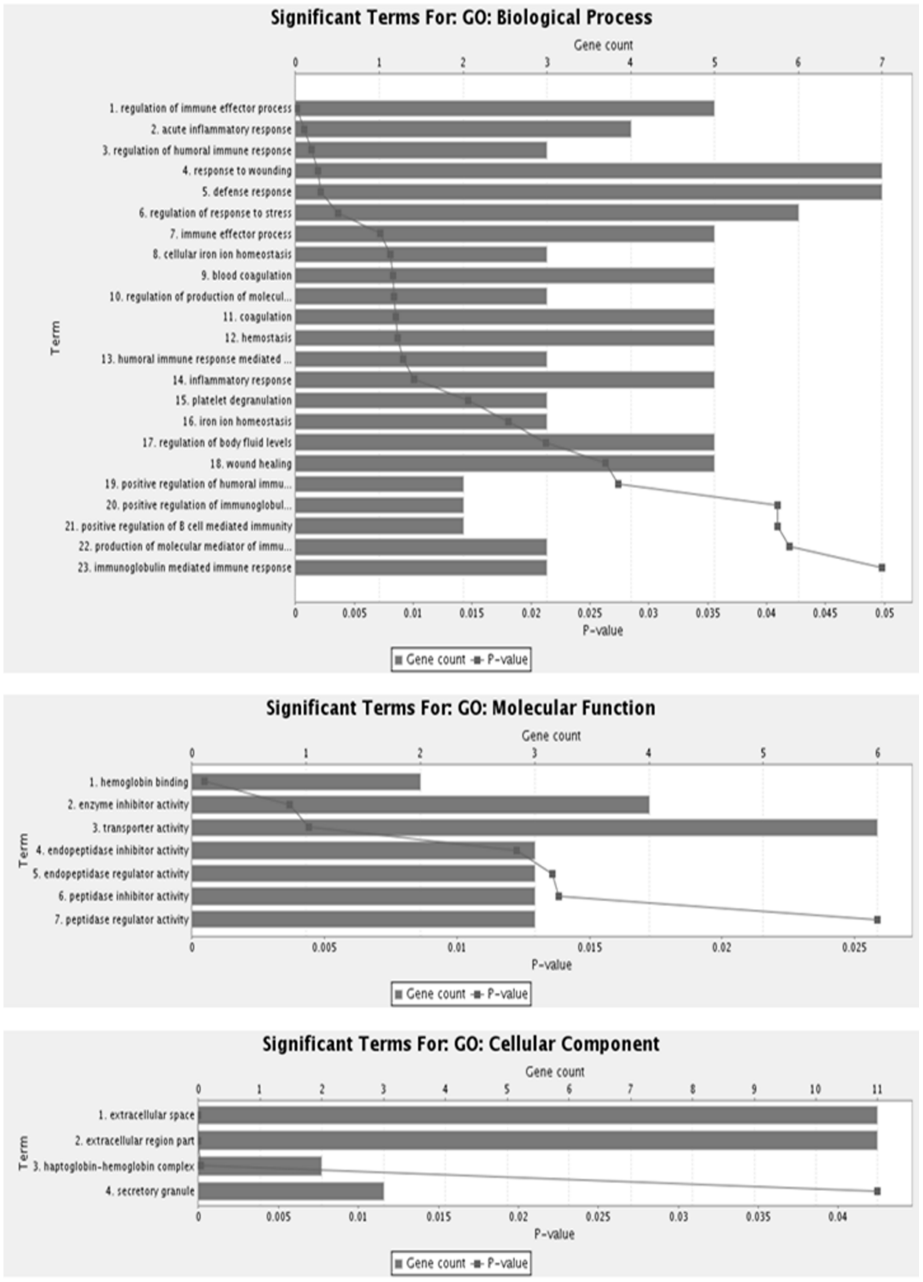
Gene ontology annotations of the proteins identified by MALDI-TOF/MS. Results were obtained using Toppgene Suite. The distribution of identified proteins according to their (A) biological processes (B) molecular functions and (C) cellular functions.

**Figure 4:**
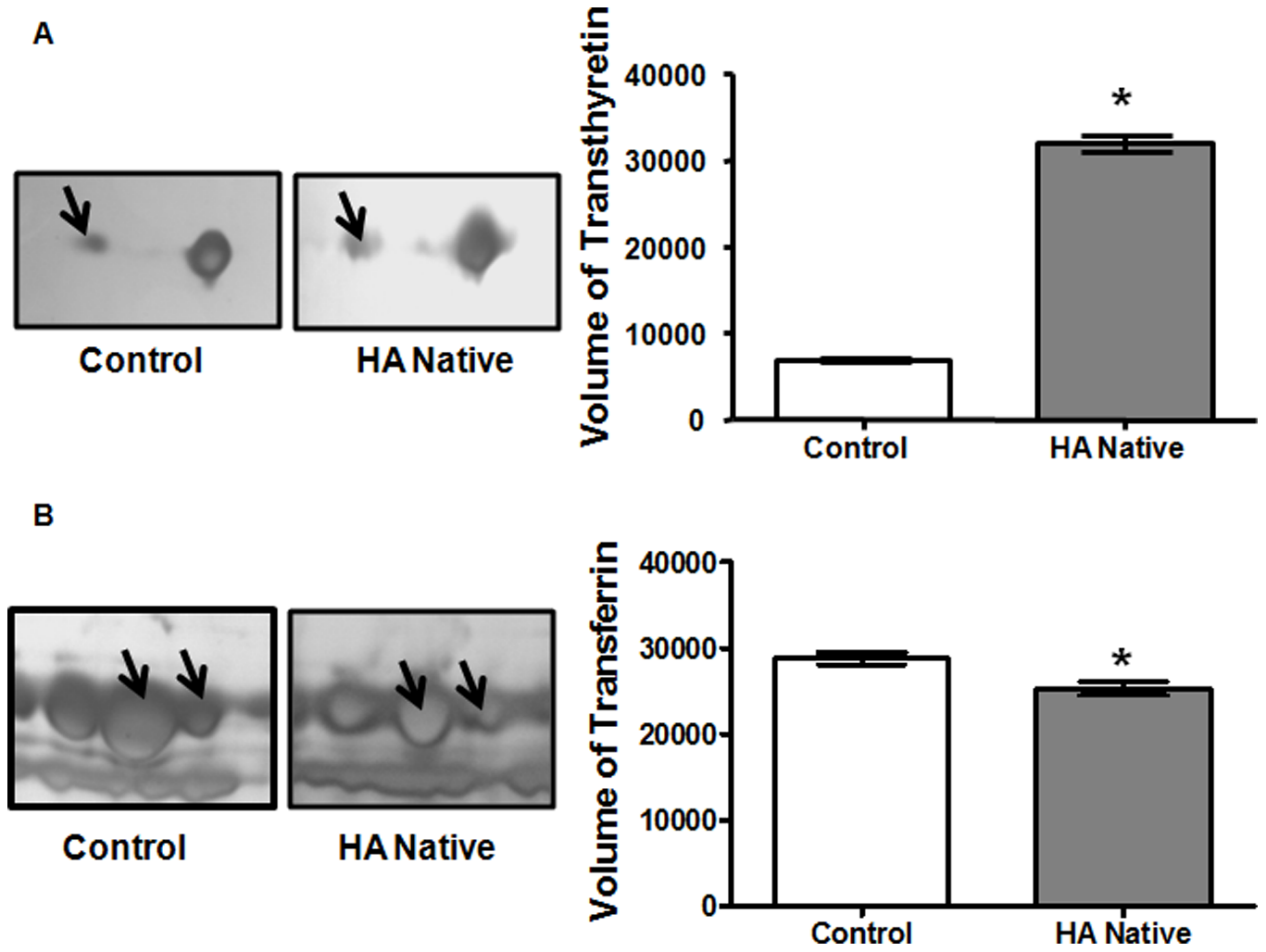
Magnified comparison maps of spot (A) 25, (B) spot 8 and 9 in the 2-DE patterns of control and HAN. Spot 25 had low expression in the control group but its expression increased in HAN. Spot 8 and 9 had high expression in the control group, but its expression decreased steadily in HAN.

**Figure 5:**
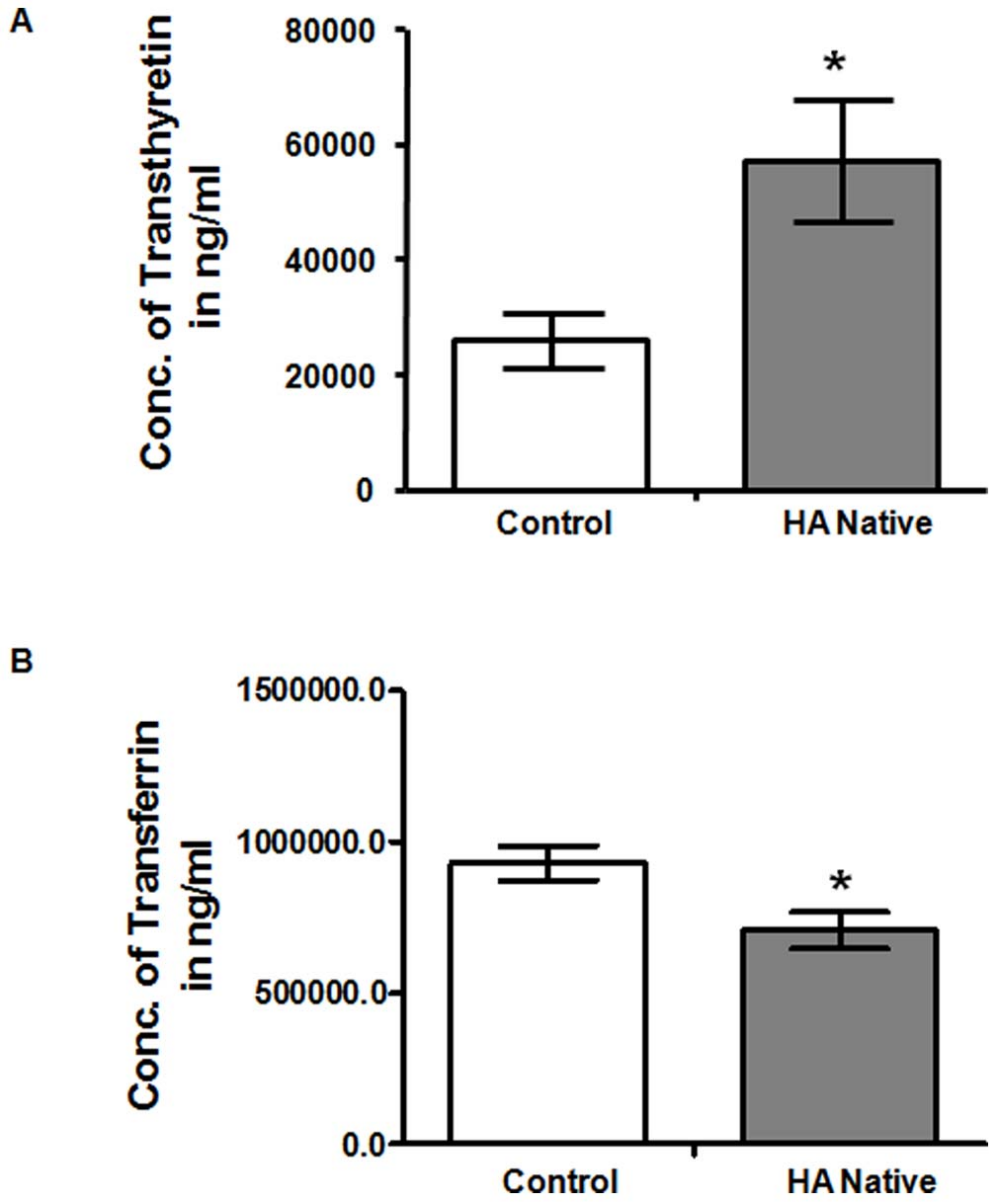
ELISA analysis of controls and high altitude natives. ELISA confirmed that the mean plasma concentration of transthyretin (A) was significantly increased and transferrin (B) was significantly decreased as compared to sea level controls (p<0.01). Data represents the Mean ± SD of three independent experiments. ‘*’ showed p<0.01 when compared to controls.

**Figure 6:**
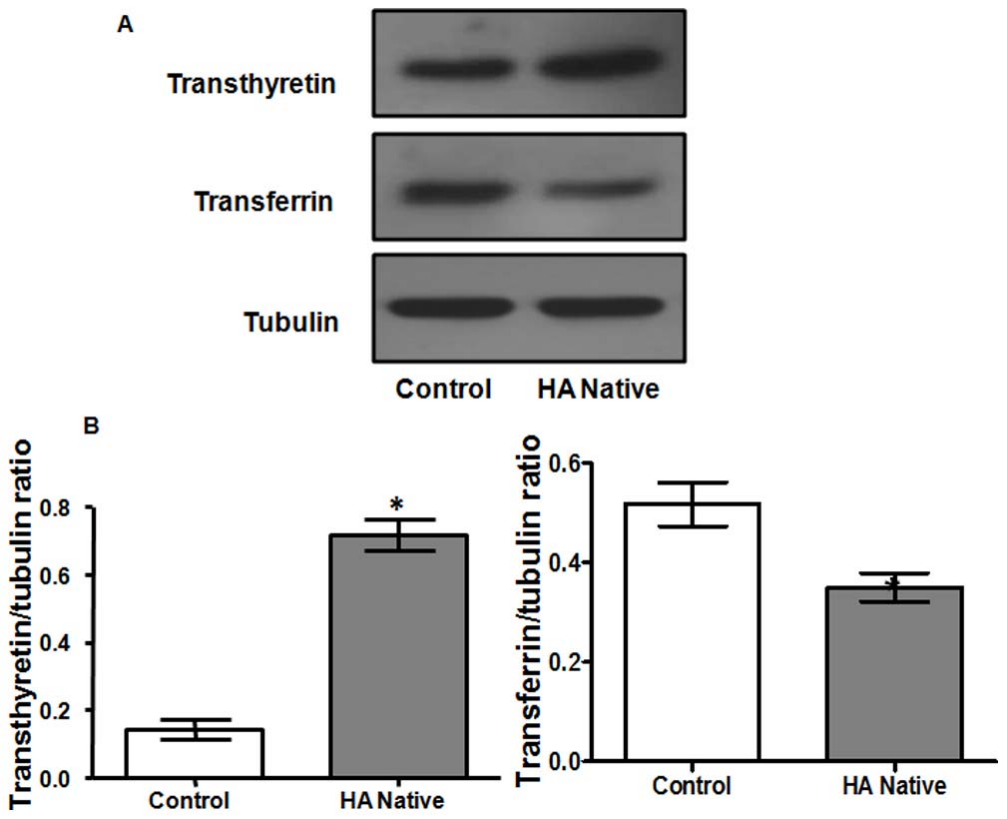
Western blot analysis of transthyretin and transferrin from plasma of controls and high altitude natives. **(A)** Blots of transthyretin and transferrin were represented along with **(B)** their respective relative optical densities (ROD). Data represents the Mean ± SD of three independent experiments. Densitometry analysis of results from western blot, indicating significant change between the two groups compared by Student’s t-test. ‘*’ showed significantly different (p<0.05) when compared to controls.

The difference of protein profiles between high altitude native and sea level control plasma was examined using 2-DE with nonlinear IPG ranging from pH 5–8. For each group, three 2-DE silver stained gels were integrated, and analyzed by Image Master 2D Platinum v.6 software, and reproducibility (>85%) was achieved. More than 700 spots were detected on each gel with molecular weight ranging from 14 to 150 kDa and pI value between 5 and 8 (Figure 2). In the comparative two-dimensional gel electrophoresis technique, a ≥50% change was considered significant and sufficient to take account of systematic errors, therefore, fold changes of ≥1.5 or ≤0.5 were significant, with a fold change value of 1 representing no difference in the proteins between the two states. Compared to normal plasma, 35 protein spots were found, of which 28 spots were up-regulated (1–7, 12–16, and 19–34), while 7 protein spots were down-regulated (8–11, 17–18 and 35). The locations of these 35 differentially expressed protein spots were marked with numbers in one representative gel shown in Fig. 2. These 35 significantly deregulated spots were successfully identified by analysis of MALDI-TOF/TOF with PMF and MS/MS followed by database searching (Figure 2, Table 1). Table 1 summarizes the identification information including mascot score, accession number, protein name, gene name, theoretical and observed pI/molecular weight value, sequence coverage, peptide match, significant fold change and protein function. Several spots were identified as the same protein, suggesting the presence of isoforms. The differentially expressed proteins listed here represent a wide range of biological categories (Figure 3). Proteins related to cellular defense mechanisms involving anti-inflammatory and antioxidant activity were the most common. When organized according to their molecular functions, several identified proteins correspond to those involved in enzymatic inhibitor (GO: 0005215) and transporter activity, (GO: 0004857). We also identified GO like haemoglobin binding (GO: 0030492) and haptoglobin-hemoglobin complex (GO: 0031838). We also categorized proteins according to their biological processes; most abundant groups of proteins corresponds to those involved in homeostatic process (G0:0007599), acute inflammatory response (GO: 0002526), inflammatory response (GO: 0006954), defense response (GO: 0006952), cellular iron ion homeostasis (GO:0006879), iron ion homeostasis response (GO:0055072), regulation of body fluid levels (GO:0050878), wound healing (GO:0042060), regulation of response to stress (GO:0080134), positive regulation of B cell mediated immunity (GO:0002714), blood coagulation (GO:007596), coagulation (GO:0050817) and immune effector process (GO:0002252). To validate the result of proteomic analysis, 2 proteins were selected for ELISA and western blotting analysis. We verified whether the expression patterns of selected proteins of transthyretin (TTR) and transferrin (TF) observed in 2-DE gels (Figure 4) paralleled those validated by ELISA and western blot analysis. ELISA analysis of 10 high altitude natives and 10 healthy controls confirmed that concentration of transthyretin (TTR) was significantly increased in the plasma of high altitude natives while the concentration of transferrin (TF) was significantly decreased (Figure 5). The mean plasma TTR concentration was 57190±10600 ng/ml (mean ± SD) in high altitude natives versus 25810±4745 ng/ml in sea level controls (p<0.01); the mean TF concentration was 706300±60210 ng/ml in high altitude natives versus 929900±57230 ng/ml in sea level controls (p<0.01). The expression patterns of both proteins transthyretin (TTR) and transferrin (TF) in plasma of high altitude natives obtained by western blot analysis (Figure 6) were in agreement with 2-DE results, so the results of ELISA analysis and western blot analysis confirmed the reliability of the proteomic analysis.

**Table 1:** List of differentially expressed plasma proteins in high altitude natives, identified by MALDI-TOF/TOF.

Spot ID	Mascot Score	SwissProt Accession No.	Protein Name	Gene Name	Theoretical pI/mass.kDa	Observed pI/mass.kDa	% Coverage	Peptide Match	SignificantFold Change	Protein Function
1	62	P02774	Vitamin D –binding protein precursor	GC	5.65/55	5.3/54.49	22	5	1.78^b^	In plasma, it carries the vitamin D sterols and prevents polymerization of actin by binding its monomers.
2	62	P02774	Vitamin D –binding protein precursor	GC	5.65/55	5.3/54.49	22	5	1.57^a^	As described earlier
3	62	P02774	Vitamin D –binding protein precursor	GC	5.65/55	5.3/54.49	22	5	1.62^a^	As described earlier
4	101	P02790	Hemopexin	HPX	5.59/72	6.5/52.38	24	8	1.92^c^	Binds heme and transports it to the liver for breakdown and iron recovery, after which the free hemopexin returns to the circulation.
5	93	P02790	Hemopexin	HPX	5.59/72	6.5/52.38	24	8	1.68^b^	As described earlier
6	93	P02790	Hemopexin	HPX	5.59/72	6.5/52.38	26	9	1.86^c^	As described earlier
7	93	P02790	Hemopexin	HPX	5.59/72	6.5/52.38	26	9	2.12^c^	As described earlier
8	144	P02787	Transferrin	TF	6.64/76	6.8/79.28	31	17	−1.93^c^	Cellular iron ion homeostasis
9	103	P02787	Transferrin	TF	6.64/76	6.8/79.28	15	8	−1.95^c^	As described earlier
10	103	P02787	Transferrin	TF	6.64/76	6.8/79.28	15	8	−1.61^a^	As described earlier
11	52	P01024	Complement C3	C3	6/184	6.98/70	8	2	−3.54^c^	Mediator of local inflammatory process
12	117	P01009	Alpha-1-antitrypsin	SERPINA1	4.95/55	5.3/46.87	3	1	1.83^c^	Inhibitor of serine proteases, Acute phase response
13	59	P00738	Haptoglobin β chain	HP	6.10/39	6.1/45.86	24	11	1.64^b^	It combines with free plasma Hb, preventing loss of iron through the kidneys and protecting the kidneys from damage by hemoglobin, while making the hemoglobin accessible to degradative enzymes
14	59	P00738	Haptoglobin β chain	HP	6.10/39	6.1/45.86	24	11	1.53^a^	As described earlier
15	59	P00738	Haptoglobin β chain	HP	6.10/39	6.1/45.86	24	11	1.58^a^	As described earlier
16	59	P00738	Haptoglobin β chain	HP	6.10/39	6.1/45.86	24	11	1.62^a^	As described earlier
17	60	P02743	Serum Amyloid P	APCS	6.12/24	5.5/26.28	15	2	−1.54^a^	It Can interact with DNA and histones and may scavenge nuclear material released from damaged circulating cells
18	90	P0C0L4	Compliment C 4A	C4A	6.99/193	6.3/30.22	25	10	−1.83^ c^	C4 plays a central role in the activation of the classical pathway of the complement system and inflammatory response
19	80	P02647	Apolipoprotein A1	APOA1	5.52/30	5.5/30.75	40	12	1.92^c^	Participates in the reverse transport of cholesterol from tissues to the liver for excretion by promoting cholesterol efflux from tissues and by acting as a cofactor for the lecithin cholesterol acyltransferase (LCAT). As part of the SPAP complex, activates spermatozoa motility
20	80	P02647	Apolipoprotein A1	APOA1	5.52/30	5.5/30.75	40	12	1.55^a^	As described earlier
21	172	P02647	Apolipoprotein A1	APOA1	5.52/30	5.5/30.75	59	20	1.8^c^	As described earlier
22	77	P00738	Haptoglobin α2 chain	HP	6.10/39	6.1/45.86	22	10	1.51^a^	As described earlier
23	94	P00738	Haptoglobin α2 chain	HP	6.10/39	6.1/45.86	22	10	1.64^a^	As described earlier
24	97	P00738	Haptoglobin α2 chain	HP	6.10/39	6.1/45.86	22	11	1.69^b^	As described earlier
25	47	P02766	Transthyretin	TTR	5.77/15	5.26/15.59	20	1	1.8^ c^	Thyroid hormone-binding protein. Probably transports thyroxine from the bloodstream to the brain
26	97	P00738	Haptoglobin α2 chain	HP	6.10/39	6.1/45.86	22	11	1.75^ c^	As described earlier
27	434	P68871	Hemoglobin beta chain	HBB	16/7.88	7.8/12	64	10	2.21^ c^	Involved in oxygen transport from the lung to the various peripheral tissues.LVV-hemorphin-7 potentiates the activity of bradykinin, causing a decrease in blood pressure
28	117	P01009	Alpha-1-antitrypsin	SERPINA1	4.95/55	5.3/46.87	3	1	1.79^b^	As described earlier
29	62	P02774	Vitamin D –binding protein precursor	GC	5.65/55	5.3/54.49	22	5	1.63^a^	As described earlier
30	62	P02774	Vitamin D –binding protein precursor	GC	5.65/55	5.3/54.49	22	5	1.51^a^	As described earlier
31	80	P02647	Apolipoprotein A1	APOA1	5.52/30	5.5/30.75	40	12	1.57^a^	As described earlier
32	80	P02647	Apolipoprotein A1	APOA1	5.52/30	5.5/30.75	40	12	1.57^a^	As described earlier
33	172	P02647	Apolipoprotein A1	APOA1	5.52/30	5.5/30.75	59	20	1.61^a^	As described earlier
34	59	P00738	Haptoglobin β chain	HP	6.10/39	6.1/45.86	24	11	1.55^a^	As described earlier
35	61	P02753	Plasma retinol binding protein	RBP4	5.27/18	5.5/23.19	61	7	−1.87^c^	Delivers retinol from the liver stores to the peripheral tissues. In plasma, the RBP-retinol complex interacts with transthyretin, this prevents its loss by filtration through the kidney glomeruli.

‘a’ denotes p<0.05. ‘b’ denotes p<0.01 and ‘c’ denotes p<0.001.

## Discussion

With increase in altitude, atmospheric pressure and the partial pressure of oxygen decrease rapidly, therefore, O_2_ availability also decreases resulting in situation termed as hypobaric hypoxia which stresses biological systems because of non-availability of steady uninterrupted supply of oxygen for mitochondrial metabolism. The degree of the adaptive responses depends on the duration of hypoxia. Short-term reduction of O_2_ supply is followed by the modification of existing proteins through phosphorylation or other post translational changes [23]. When low O_2_ concentration is sustained, longer adaptive alterations of gene expression take place and finally chronic hypoxia results in cell death when the adaptive mechanisms are exhausted [24]. Short-term hypoxia can prime cells or organs to increase their tolerance to hypoxia [25], [26] for example through down-regulation of energy demanding pathways to reduce ATP requirements [27]–[29]. Another compensatory factor is the up-regulation of protective components against reactive oxygen species generated during reoxygenation. Since hypoxia represents a major challenge for cells, subsequent changes of gene expression, protein degradation, and other post-translational modifications should result in alteration of the proteome [30].

In this study, we used the comparative proteomics technology to demonstrate the characteristic alterations of the plasma proteins in high altitude natives after the depletion of two high abundant-proteins (albumin and IgG). The expression levels of 35 protein spots, which were identified by MALDI-TOF/TOF, showed significant changes in high altitude natives compared with controls. Several identified proteins related to oxidative and cellular defense mechanisms involving anti-inflammatory and antioxidant activity were the most common. The identified biological/cellular functions represent relevant targets for the adaptation processes under the influence of hypobaric hypoxia. The relationships of these proteins with high altitude natives are elucidated in the following section.

### Regulation of Up-regulated Proteins in High Altitude Natives

Vitamin D-binding protein (VDBP, spot no: 1–3, 29 and 30) belongs to the albumin superfamily of binding proteins that includes albumin, α-albumin, and α-fetoprotein. Besides its specific sterol binding capacity, VDBP exerts several other important biological functions such as fatty acid transport, macrophage activation [31] and chemotaxis [32]. In addition, VDBP also mediates inflammatory and immunoregulatory activities in response to environmental challenges. Given the diverse and physiologically important roles of VDBP, down-regulation could contribute to a variety of health concerns [33], [34]. Reduced levels of DBP have been observed in trauma patients who go on to develop organ dysfunction and sepsis, with complete depletion of free DBP in septic shock and hepatic necrosis being associated with a fatal outcome [35]–[38]. In this study, the high level of VDBP in high altitude natives may reflect its role as a scavenger protein or of protection against inflammation and might contribute to the mechanism of adaptation at high altitude hypoxia.

Hemoproteins undergo degradation during hypoxic/ischemic conditions, but the pro-oxidant free heme that is released cannot be recycled and must be degraded. The extracellular heme associates with its high-affinity binding protein, hemopexin (HPX). Hemopexin (spot no: 4–7) is the important constituent of iron homeostasis system, regulating cellular iron levels. It is mainly expressed in liver, and belongs to acute phase reactants, the synthesis of which is induced after inflammation. Heme is potentially highly toxic because of its ability to intercalate into lipid membrane and to produce hydroxyl radicals. The binding strength between heme and HPX, and the presence of a specific heme-HPX receptor able to catabolize the complex and to induce intracellular antioxidant activities, suggest that hemopexin is the major vehicle for the transportation of heme in the plasma, thus preventing heme-mediated oxidative stress and heme-bound iron loss. A previous study has reported that genetic deletion of HPX significantly increases the severity of the brain damage from ischemic stroke, that heme–HPX protects cells and particularly neurons against both heme- and oxidative stress-induced toxicity [39]. In this study, we found that the plasma concentration of HPX was increased significantly, suggesting the most important physiological role as an antioxidant in high altitude natives by maintaining the tight balance between free and bound heme.

A further interesting protein identified in high altitude natives was alpha-1- antitrypsin (SERPINA 1, spot no: 12 and 28). It plays an important role in wound-healing [40], inhibiting plasmin and activating plasminogen and thrombin, and also inhibits haematopoietic stem cell mobilization in bone marrow [41]. Further, in vitro studies showed an increase of lipopolysaccharide-mediated macrophage activation and anti-inflammatory effects on B-cells, demonstrating an additional role in immune regulation [42]. Deficiencies of alpha- 1-antitrypsin resulted in elastase-induced tissue damage, such as skin hyperextensibility [43], chronic obstructive pulmonary disease and liver disease in humans [42]. Major functions of the protein are to inactivate neutrophil elastase and other proteases to maintain a protease-antiprotease balance [44], to protect connective tissue of the lung from degradation by elastase, and to prevent the destruction of pulmonary extracellular matrix. It was reported that progression of pulmonary hypertension was associated with increased serine elastase activity. Moreover, administration of inhibitors of serine elastase to monocrotaline-exposed rats reduced changes of pulmonary hypertension [45]. In addition to elastase inhibition, alpha-1-antitrypsin could also prevent lung endothelial cell apoptosis by inhibiting caspase-3 activity [46]. It was reported that decrease of alpha-1-antitrypsin might induce to increased apoptosis of endothelial cell, which could result in proliferation of apoptosis-resistant endothelial cell [47] and arteriolar occlusion [48]. The proliferating endothelial cells often form plexiform lesions and may also contribute to vascular wall thickening. Impairment of vascular and endothelial homeostasis was thought to play a major role in the initiation and development of pulmonary arterial hypertension [49], [50]. These functions of alpha-1-antiproteinase make it an interesting protein that was up-regulated in high altitude natives.

Another protein whose expression was different in plasma from high altitude natives compared with that from sea level controls was haptoglobin. Haptoglobin (HP, spot no: 13–16, 22–24 and 26) is an acute phase reactant protein that functions as an antioxidant by virtue of its ability to bind to haemoglobin [51] and thereby to prevent the oxidative tissue damage that may be mediated by free haemoglobin [52]–[54]. The importance of this protective mechanism has been demonstrated in haptoglobin knockout mice in which a marked increase in oxidative tissue damage develops in response to hemolysis [51], [55], [56]. Besides its scavenging and inflammatory response functions, Hp has been shown to be involved in the regulation of epidermal cell transformation [57], immune suppression in cancer [58], and angiogenesis [59]. Haptoglobin consists of two different polypeptide chains: the α and β chains [60]. The β chain (40 kDa) is heavier than the α chain and is identical in all the Hp types [61]. In our study we detected nine haptoglobin spots with an apparent experimental mass of 45 kDa, which suggests the presence of the haptoglobin β chain. The expression level of haptoglobin β is regulated by several cytokines, including IL-1, IL-6, TNF-α, and TGF-β. Plasma expression of haptoglobin β chain was significantly increased in high altitude natives compared with that from healthy sea level controls, suggesting the protective role as an antioxidant against the oxidative damage caused by hypobaric hypoxia.

Apolipoprotein A-I (APOA1, spot no: 19–21 and 31–33) was also identified in high altitude natives by comparison with controls. It belongs to the APOA1/A4/E protein family and is primarily produced in the liver and the intestine. APOA1 can be found in the extracellular space and, being a structural component of high density lipoprotein (HDL), takes part in cholesterol absorption. APOA1 up-regulation is associated with breast and lung cancer as suggested elsewhere [62]. APOA1 is also linked to antioxidant function that is proposed to be involved in its vasculoprotective activity, apparently by complexing with paraoxonase [63]. Interestingly, studies provide new evidence supporting the notion that HDL plays a protective role in the lung. ABCA1, which interacts with lipid-poor APOA1, was earlier shown to be essential for maintaining normal lipid composition and architecture of the lung as well as respiratory physiology [64]. There is emerging evidence that, APOA1 plays a critical role in protecting pulmonary artery and airway function as well as preventing inflammation and collagen deposition in the lung [65]. More recently, proteomic studies revealed the anti-inflammatory role of APOA1 in HAPE patients [21]. Here we report APOA1expression was up-regulated in high altitude natives suggesting the anti-inflammatory role of APOA1.

Another reported negative acute phase protein identified in high altitude natives was transthyretin (TTR, spot no: 25). TTR is a multifunctional protein which interacts with several molecules of biological interest. Both its interaction with RBP and its binding property for thyroid hormones have been well established [66], [67] and recently further interactions and functions have been described [68]. Traditionally, TTR has been regarded as a biomarker for nutritional status as it is synthesized in the liver in response to nutritional supply. TTR plasma levels have thus been used as sensitive biochemical parameters of subclinical malnutrition, as both the adequacy of protein as well as energy intakes are reflected in its plasma levels. Plasma levels of TTR however, are also affected by acute and chronic diseases associated with an acute-phase response. Under these conditions, liver activity is concentrated on the synthesis of acute-phase response proteins, resulting in a drop in visceral proteins [66], [69]. A truncated variant of TTR has been described as a biomarker for ovarian cancer [70], indicating a close interplay between nutritional status, inflammation and possibly the occurrence of cancer [71]. Previous work on TTR null mice indicated the potential neuroprotective role of TTR in cerebral ischemia in absence of a full heat-shock response and contributes to control neuronal cell death, edema and inflammation, thereby influencing the survival of endangered neurons in cerebral ischemia [72]. A persistent low level of serum transthyretin is predictive of lethality, whereas increased levels were associated with improved ventilator performances [73]. Here in this study, TTR was found to be up-regulated in high altitude natives. The mean plasma TTR concentration was increased to 57190±10600 ng/ml (Mean ± SD) in high altitude natives as compared to sea level controls that was 25810±4745 ng/ml (p<0.01). Taken together, we can conclude that the overexpression of TTR possibly improves ventilatory function in high altitude natives.

Hemoglobin, the key component of oxygen storage and regulation system, is widely distributed in all living organism. It is unique in its ability to adapt to a wide range of environmental conditions [74]. High altitude hypoxia affects oxygen transport properties of haemoglobin and alters oxygen affinity by several mechanisms. All modifications adopted by animals appear to optimize both arterial oxygen loading and peripheral unloading [75]. The hemoglobin affinity for oxygen allows rapid adjustments of oxygen binding and release since the process is far less energy demanding than an increase in cardiac output. This adaptation has been attributed to changes in the primary structure of globin chains, which modulates oxygen uptake and delivery to the tissues. In this study, we found that haemoglobin beta chain (HBB, spot no: 27) was up-regulated in high altitude natives, providing the essential information for elucidating the possible roles of HBB in adapting to high altitude environment.

### Regulation of Down-regulated Proteins in High Altitude Natives

Iron plays a central role in protecting the organism from hypobaric hypoxia, as it is incorporated in the newly synthesized haemoglobin throughout erythropoiesis. The findings of the present study show that, in humans, this essential adaptive response to the hypobaric hypoxia is associated with a net loss in the iron content in the plasma, as indicated by the down-regulation of the iron protein that is transferrin (TF, spot no: 8–10) which is the major transporter of iron from its storage sites to the bone marrow [76], [77]. Transferrin is an important constituent of the iron homeostasis system, regulating cellular iron levels. The molecular mechanisms underlying TF down-regulation is poorly understood. In addition to iron-mediated posttranscriptional regulation, TF expression is also subject to HIF-1– mediated transcriptional control, but the finding of increased HIF-1 mRNA levels during high-altitude exposure does not offer a satisfactory molecular basis for the down-modulation of TF because evidence from cell culture studies indicates that HIF 1 induces TF transcription under hypoxic conditions [78], [79]. However, whatever the mechanism may be, when considering the role of TF in iron uptake, lower TF expression are consistent with the loss of iron from the plasma of natives exposed to high altitude, which might in turn lead to an overload of iron [80], [81]. In the current study, the mean plasma TF concentration was decreased to 706300±60210 ng/ml (Mean ± SD) in high altitude natives as compared to sea level controls that was 929900±57230 ng/ml (p<0.01), providing the new insight into the molecular aspects of iron-oxygen interactions in response to hypobaric hypoxia.

The normal circulating plasma protein serum amyloid P-component (APCS, spot no: 17) occurs as a non-fibrillar constituent of all amyloid deposits, accounting for up to 15% of their mass. SAP is a member of the pentraxin protein family which includes C-reactive protein. Human SAP is secreted and catabolised only by hepatocytes, and consists of five identical noncovalently associated subunits, each with a molecular mass of 25 kDa, which are non-covalently associated in a pentameric disc-like ring [82]. SAP is a calcium dependent ligand binding protein, which binds to DNA and chromatin [83] and to all known types of amyloid fibrils accounting for its specific accumulation in amyloid deposits. No deficiency or polymorphism of SAP has been described and it has been stably conserved in evolution. Physiological functions of SAP, supported by studies in knockout mice, may include regulation of DNA and chromatin clearance and a contribution to innate host resistance to a range of infections [84]. The SAP molecule is highly resistant to proteolysis and its binding to amyloid fibrils in vitro protects them against proteolytic degradation [85]. A contribution of SAP to amyloidogenesis in vivo has been conforrmed in SAP knockout mice [86]. Even though SAP was discovered nearly four decades ago and has been intensely studied, the exact function of this homopentameric protein still remains elusive. However, the role serum amyloid P component plays in amyloid plaque formation and stability has been well documented which has recently led to it becoming the target of a new anti-Alzheimer’s therapy [87]. In the present study, serum amyloid P concentration was down-regulated in the plasma of high altitude natives.

The complement system is important for cellular integrity and tissue homeostasis. It plays a major physiological role in body defense as part of the innate and adaptive immune systems. Complement activation mediates the removal of microorganisms and the clearance of modified self cells, such as apoptotic cells. Complement regulators control the spontaneously activated complement cascade and any disturbances in this delicate balance can result in damage to tissues and in autoimmune disease [88], [89]. In addition, complement components C3 and C4a have been implicated as biomarkers of idiopathic pulmonary hypertension [90], [91]. Recent study provides new evidence that deficiency of C3 attenuates chronic hypoxia-induced pulmonary hypertension in mice [92]. In the present study, the level of the complement component C3 (spot no: 11) and C4A (spot no: 18) was also found to be down-regulated in plasma from high altitude natives. This result is in accordance with previous observations in the literature indicating the important adaptive role of these two proteins in high altitude natives.

Dietary vitamin compounds transported as retinyl esters by chmylomicrons are rapidly taken up by liver parenchymal cells, then transferred and stored as holoRBP (conveying retinol) in stellate cells, representing until released into the bloodstream the major reservoir (up to 80–90%) of total vitamin A [93]. Retinol binding protein (RBP 4, spot no: 35) is a vitamin A transport protein that acts as an adipokine when secreted from adipose tissue. RBP 4 down-regulates GLUT4 [94], the insulin-activated glucose transporter responsible for translocation of glucose into both muscle and fat cells [95], and has also recently been shown to induce expression and secretion of pro-inflammatory cytokines in primary human macrophages known to induce insulin resistance [96]. Recent studies have demonstrated that during the onset of a stress reaction of medium severity, the reduction by half of RBP plasma levels releases more retinol in free form into the extracellular space in amounts corresponding to about 10 times the normal free concentration [97]. Under physiological conditions, RBP binds transthyretin (TTR) to prevent glomerular filtration of low molecular weight RBP in the kidneys. The RBP/TTR molar ration provides an indirect way to indicate marginal vitamin A deficiency. In this study, we also found that RBP was down-regulated in the plasma of high altitude natives. The low plasma concentration of RBP might result from the declining synthesis of RBP in livers.

### Concluding Remarks

In summary, proteomic analysis of plasma from high altitude natives allowed the confident identification of several differentially expressed proteins in comparison to normal plasma. The proteomic information derived from the proteins detected in an increased or decreased level to determine if their protein-expression signature could predict an adaptive response to hypobaric hypoxia in the plasma of high altitude natives. Interpreting the biological significance of these differences in protein expression is not an easy task but according to literature and current findings these proteins had a relatively high abundance in the plasma, and they all play a positive anti-inflammatory role. The results show that there is some adaptive mechanism that sustains the inflammation balance and the homeostasis of the body in high altitude natives, thereby facilitating physical activity under extreme conditions. Importantly, we report 35 proteins with altered proteomic patterns in the plasma of high altitude natives, opening the door to further identification of mechanisms involved in hypobaric hypoxia and to their potential assessment as novel protein markers.
